# Zika virus in semen: a prospective cohort study of symptomatic travellers returning to Belgium

**DOI:** 10.2471/BLT.17.181370

**Published:** 2017-12-01

**Authors:** Ralph Huits, Birgit De Smet, Kevin K. Ariën, Marjan Van Esbroeck, Emmanuel Bottieau, Lieselotte Cnops

**Affiliations:** a Department of Clinical Sciences, Institute of Tropical Medicine, Kronenburgstraat 43/3, B-2000, Antwerp, Belgium.; b Department of Biomedical Sciences, Institute of Tropical Medicine, Antwerp, Belgium.

## Abstract

**Objective:**

To prospectively monitor Zika viral loads in semen from Belgian travellers with confirmed Zika virus infection, who returned from the Americas during the 2016 Zika virus epidemic.

**Methods:**

We recruited symptomatic travellers consulting our clinic and we confirmed infection with either reverse-transcriptase (RT) polymerase chain reaction (PCR) assay or virus neutralization test. The participants produced semen samples weekly, either at the clinic or at home. For the initial sample, the laboratory staff did a microscopy analysis if they received the sample within an hour of production. Using RT–PCR, we monitored Zika virus ribonucleic acid (RNA) loads in semen until we obtained two negative results.

**Findings:**

We detected Zika virus RNA in nine of 15 participants’ semen, one of whom was vasectomized. The median time to loss of RNA detection in semen was 83 days after symptom onset (95% confidence interval, CI: 57−108). The longest duration of viral shedding in semen before obtaining the first negative RT–PCR result was 144 days after symptom onset. All of the 11 participants, for whom we microscopically analysed their semen, had presence of leukocytes, 10 showed haematospermia and six showed oligospermia. These abnormalities occurred irrespective of Zika virus detection in semen.

**Conclusion:**

The majority of men in our study had detectable Zika virus RNA in their semen. We recommend that semen from Zika virus-infected men should be analysed with RT–PCR and that health professionals should advise infected men, even if they are vasectomized, about current recommendations for prevention of sexual transmission of the virus.

## Résumé

**Objectif:**

Assurer un suivi prospectif des charges virales du virus Zika dans le sperme de voyageurs belges dont l'infection à virus Zika a été confirmée et qui sont rentrés du continent américain lors de l'épidémie du virus Zika de 2016.

**Méthodes:**

Nous avons recruté des voyageurs symptomatiques qui se sont rendus dans notre centre de consultation et avons confirmé l'infection à l'aide d'un test basé sur l'amplification en chaîne par polymérase après transcription inverse (RT-PCR) ou d'un test de neutralisation du virus. Les participants ont produit un échantillon de sperme par semaine, chez eux ou dans le centre. Pour le premier échantillon, le personnel de laboratoire a procédé à une analyse au microscope si l'échantillon était fourni dans l'heure de production. À l'aide d'une RT-PCR, nous avons suivi les charges d'acide ribonucléique (ARN) du virus Zika dans le sperme jusqu'à l’obtention de deux résultats négatifs.

**Résultats:**

Nous avons détecté l'ARN du virus Zika dans le sperme de 9 des 15 participants, l'un d'entre eux ayant subi une vasectomie. Le délai médian pour que l'ARN ne soit plus détecté dans le sperme était de 83 jours après l'apparition de symptômes (intervalle de confiance, IC, à 95%: 57-108). La plus longue durée d'excrétion virale dans le sperme avant l'obtention du premier résultat négatif de la RT-PCR était de 144 jours après l'apparition de symptômes. La présence de leucocytes a été détectée chez les 11 participants dont nous avons analysé le sperme au microscope; dix d'entre eux souffraient d'hématospermie et six d'oligospermie. Ces anomalies peuvent survenir indépendamment de la présence du virus Zika dans le sperme.

**Conclusion:**

Nous avons détecté l'ARN du virus Zika dans le sperme de la majorité des hommes de notre étude. Nous recommandons que le sperme des hommes infectés par le virus Zika soit analysé à l'aide d'une RT-PCR et que les professionnels de santé communiquent les recommandations actuelles concernant la prévention de la transmission du virus par voie sexuelle aux hommes infectés, y compris ceux ayant subi une vasectomie.

## Resumen

**Objetivo:**

Supervisar de forma prospectiva las cargas víricas del Zika en el semen de viajeros belgas con una infección confirmada del virus del Zika, que regresaban del continente americano durante la epidemia del virus del Zika de 2016.

**Métodos:**

Reclutamos a viajeros sintomáticos que consultaban nuestra clínica y confirmamos la infección con las pruebas de reacción en cadena de la polimerasa de transcriptasa inversa (RT-PCR) o con la prueba de neutralización del virus. Los participantes produjeron muestras de semen de forma semanal, en la clínica o en sus casas. Para la muestra inicial, el personal del laboratorio realizaba un análisis de microscopio si recibía las muestras durante la hora de producción. Usando la RT-PCR, supervisamos las cargas de ácido ribonucleico (RNA) del virus del Zika en el semen hasta que obtuvimos dos resultados negativos.

**Resultados:**

Detectamos RNA del virus del Zika en el semen de nueve de quince de los participantes, uno de los cuales se había hecho una vasectomía. El promedio de tiempo hasta la detección de pérdida de RNA en el semen fue de 83 días tras el comienzo de los síntomas (intervalo de confianza (IC) del 95%: 57−108). El periodo máximo de eliminación del virus en el semen antes de obtener el primer resultado negativo de la RT-PCR fue de 144 días tras el comienzo de los síntomas. Los once participantes cuyo semen se analizó con el microscopio presentaron leucocitos, diez de ellos mostraron hematospermia y seis oligospermia. Estas anomalías se produjeron independientemente de la detección del virus del Zika en el semen.

**Conclusión:**

La mayoría de los hombres del estudio tenían RNA del virus del Zika detectable en el semen. Se recomienda que el semen de los hombres infectados por el virus del Zika se analice con RT-PCR y que los profesionales de la salud aconsejen a los hombres infectados, incluso si se han hecho la vasectomía, sobre las recomendaciones actuales para la prevención de la transmisión sexual del virus.

## ملخص

**الغرض:**

إجراء رصد تنبؤي لحمل فيروس زيكا في السائل المنوي لدى المسافرين البلجيكيين الذين تأكدت إصابتهم بعدوى فيروس زيكا والعائدين من الأمريكيتين خلال عام 2016 حينما انتشر وباء فيروس زيكا بالمنطقة.

**الطريقة:**

استعنا بمسافرين ظهرت لديهم أعراض المرض، مع استشارة العيادة الطبية التابعة لنا وتأكدنا من إصابتهم بالعدوى إما من خلال تحليل تفاعل البوليميراز المتسلسل (PCR) والمستنسخة العكسية (RT) أو اختبار تحييد الفيروس. وكان المشاركون ينتجون عينات من السائل المنوي أسبوعيًا، إما في العيادة الطبية أو في منازلهم. وكان العاملون في المختبر يجرون تحليلاً بالمجهر للعينة الأولية في حالة استلامها خلال ساعة من إنتاجها. وقد رصدنا كميات حمل فيروس زيكا اعتمادًا على الحمض النووي الريبوزي (RNA) في السائل المنوي باستخدام تفاعل البوليميراز المتسلسل والمستنسخة العكسية (RT–PCR) حتى حصلنا على نتيجتين سلبيتين.

**النتائج:**

اكتشفنا وجود الحمض النووي الريبوزي (RNA) في تسع عينات من بين 15 عينة من السائل المنوي للمشاركين، حيث كانت إحدى الحالات قد خضعت لجراحة قطع القناة المنوية. وكان متوسط الفترة الزمنية التي مرت لحين الكشف عن خلو السائل المنوي من الحمض النووي الريبوزي (RNA) قد بلغ 83 يومًا بعد ظهور العرض (بنسبة أرجحية مقدارها 95‏%: 57-108). وبلغت أطول مدة لإفراز الفيروس في السائل المنوي قبل الحصول على أول نتيجة سلبية لتفاعل البوليميراز المتسلسل والمستنسخة العكسية (RT–PCR) 144 يومًا بعد ظهور العرض. وقد ظهرت كرات دم بيضاء في العينات الخاصة بجميع المشاركين الذين أجرينا تحليلاً بالمجهر للسائل المنوي لهم والبالغ عددهم 11 مشاركًا، وظهرت كذلك لدى 10 منهم علامات الإصابة بتدمي المني، بينما ظهرت لدى ستة منهم علامات الإصابة بنقص الحيوانات المنوية. ولم يتم الربط بين ظهور تلك الحالات من الخلل واكتشاف فيروس زيكا في السائل المنوي لديهم.

**الاستنتاج:**

إن معظم الرجال المشاركين في الدراسة التي أجريناها قد ظهر في السائل المنوي لديهم الحمض النووي الريبوزي (RNA) لفيروس زيكا على نحو يمكن اكتشافه. ونوصي بوجوب تحليل السائل المنوي للرجال المصابين بعدوى فيروس زيكا باستخدام تفاعل البوليميراز المتسلسل والمستنسخة العكسية (RT–PCR)، ونوصي أيضًا بوجوب تقديم النصيحة للرجال المصابين بالعدوى من جانب المختصين في مجال الصحة – حتى إذا كان المصابون قد خضعوا لجراحة قطع القناة المنوية – وذلك بشأن التوصيات المتوفرة حاليًا للوقاية من انتقال الفيروس بالاتصال الجنسي.

## 摘要

**目的:**

旨在对精液中携带寨卡病毒的比利时旅客进行前瞻性监测。这些旅客在 2016 年寨卡病毒流行期间从美洲返回比利时，被确诊携带寨卡病毒。

**方法:**

我们通过咨询诊所招募有症状的旅客，同时我们通过逆转录-聚合酶链反应 (RT–PCR) 分析或者病毒中和测试，确认感染。参与者每周在诊所或者在家提供一次精液样本。对于初始样本，实验室工作人员若在一小时之内获取样本，即对其进行显微分析。我们使用逆转录-聚合酶链反应 (RT–PCR) 方法对精液携带的寨卡病毒核糖核酸 (RNA) 进行了监测，两次监测，结果均显示阴性。

**结果:**

在 15 名参与者中，我们发现 9 名参与者的精液中有寨卡病毒核糖核酸 (RNA)，其中一名参与者做过输精管结扎手术。精液中核糖核酸 (RNA) 检测量平均减少时间是症状发作后 83 天（95% 的置信区间，CI: 57?108）。在获得首个阴性逆转录-聚合酶链反应 (RT–PCR) 结果之前，精液中病毒脱落的最长持续时间为症状发作后 144 天。在我们对其精液进行显微分析的 11 名参与者中，全部精液都存在白细胞，10 例呈现血精，6 例呈现精子减少。无论在精液中是否检测出寨卡病毒，均出现这些异常情况。

**结论:**

在我们的研究中，我们在大多数男性的精液中检测到了寨卡病毒核糖核酸 (RNA)。我们建议应该使用逆转录-聚合酶链反应 (RT–PCR) 方法分析寨卡病毒感染者的精液。医护专业人员应该向男性感染者（即使是做过输精管结扎手术的男性）提供关于预防病毒通过性行为传播的建议。

## Резюме

**Цель:**

Провести проспективный мониторинг вирусной нагрузки в сперме у бельгийских путешественников с подтвержденной вирусной инфекцией Зика, которые вернулись из Северной и Южной Америки во время эпидемии вируса Зика 2016 года.

**Методы:**

К исследованию были привлечены путешественники с клиническими симптомами, консультирующиеся в нашей клинике, и наличие инфекции у них было подтверждено с помощью метода полимеразной цепной реакции (ПЦР) с обратной транскриптазой (ОТ) или теста на реакцию нейтрализации вируса. Еженедельно в клинике или на дому у участников получали образцы спермы. Сотрудники лаборатории проводили микроскопический анализ исходного образца, если этот образец был ими получен в течение часа после отбора. С помощью метода ОТ-ПЦР мы контролировали нагрузку рибонуклеиновой кислоты (РНК) вируса Зика в сперме до получения двух отрицательных результатов.

**Результаты:**

Мы обнаружили РНК вируса Зика в девяти из 15 образцов спермы участников, один из которых был вазэктомизирован. Среднее время потери обнаружения РНК в сперме составляло 83 дня после начала проявления симптомов (95%-й доверительный интервал, ДИ: 57–108). Самая длинная продолжительность выделения вируса в сперме до получения первого отрицательного результата ОТ-ПЦР составила 144 дня после начала проявления симптомов. У всех 11 участников, для которых мы провели микроскопический анализ спермы, в образцах были обнаружены лейкоциты, у 10 из них — гематоспермия, а у шести — олигоспермия. Эти патологии встречались независимо от обнаружения вируса Зика в сперме.

**Вывод:**

У большинства мужчин в ходе нашего исследования в сперме была обнаружена РНК вируса Зика. Рекомендуется использовать метод ОТ-ПЦР для анализа спермы, полученной от зараженных вирусом Зика пациентов, а также проводить силами медицинских работников консультации для инфицированных мужчин, даже подвергнутых вазэктомии, относительно текущих рекомендаций по профилактике половой передачи вируса.****

## Introduction

Zika virus infection in humans may result in a mild disease characterized by rash, fever, arthralgia and conjunctivitis.^1^^,^^2^ However, since its introduction in the Americas in 2015, the Zika virus has been found to cause congenital brain abnormalities and Guillain–Barré syndrome.^3^ A recent study showed that for pregnant women having symptoms or tested positive for Zika virus in the first trimester, 22 (8%) out of the 276 completed pregnancies had Zika virus-associated birth defects. For second and third trimesters, the numbers were 36 (5%) out of 726 and 20 (4%) out of 494, respectively.^4^ Therefore, the Zika virus has emerged as a public and reproductive health concern.^5^

The Zika virus, which belongs to the genus *Flavivirus*, is primarily transmitted by *Aedes* mosquitoes in endemic areas, but the virus can also be transmitted from person-to-person via sexual encounter.^6^ Evidence exists that people who had not resided in or travelled to areas with arthropod-borne Zika virus transmission have developed the disease after oral, vaginal or anal sexual intercourse with Zika virus infected partners.^6^^–^^8^ Sexual transmission by asymptomatic men has been reported in a couple seeking assisted reproduction treatment and in a woman with a travelling male sex partner.^9^^,^^10^ The longest documented interval between a man’s onset of symptoms and sexual transmission to a woman is 44 days.^11^

Using reverse-transcriptase (RT) polymerase chain reaction (PCR) assay, researchers have detected Zika virus ribonucleic acid (RNA) in vaginal secretions and semen.^12^^–^^15^ Studies have reported viral shedding in semen beyond 188 days after symptom onset.^16^ Several studies have assessed Zika virus persistence in semen after acute infection in endemic settings.^13^^,^^17^^,^^18^ In a study from Puerto Rico, 56% (31/55) of semen samples from infected men contained viral RNA. The estimated median time until RNA was undetectable in the men’s semen was 34 days after symptom onset (95% CI: 28–41) and 95% of semen samples were virus negative after 81 days (95% CI: 64–98).^13^ A prospective study from French Guiana found viral RNA in 67% (8/12) of semen samples from infected men.^17^ The authors concluded that the average persistence of RNA in semen was 26 days, although the intervals between detection and follow-up sampling were irregular and large.^17^ A report on 15 infected men from the French oversea territory Guadeloupe, showed that 11 (73%) had viral RNA in semen.^18^ One study documented viral RNA in semen for 12 out of 23 (52%) British travellers.^19^ Because of loss to follow-up, the study could only report time to viral clearance in semen for four patients, which ranged from 70 to 132 days after symptom onset.

Persistence of Zika virus in immune-privileged sites, such as the male reproductive system, may differ among populations depending on ethnic backgrounds, ongoing vector-borne transmission of the virus and previous exposure to other flaviviruses. Therefore, knowledge of the incidence and kinetics of Zika virus in semen is important for assessing the probability of viral sexual transmission. Here we present the results from a cohort study that prospectively monitored viral loads in semen from Belgian travellers with confirmed Zika virus infection returning from the Americas.

## Methods

From February 2016 to May 2017, we tested all travellers who consulted the Institute of Tropical Medicine Antwerp, Belgium, with symptoms matching the European Centers for Disease Control clinical case definition for Zika virus infection – that is, maculopapular rash with or without fever, and painful joints or muscles or non-purulent conjunctivitis^20^ – for Zika virus.

To diagnose Zika virus infection, we first used the anti-Zika virus Immunoglobulin (Ig) M and IgG enzyme linked immunosorbent assay (ELISA; Euroimmun AG, Lübeck, Germany) according to the manufacturers’ exact instructions. We confirmed positive or equivocal ELISA results the same week with a non-commercial Zika virus neutralization test on refrigerated serum. To detect viral RNA, we performed commercial available Zika virus-specific RT–PCR (RealStar® Zika Virus RT–PCR Kit, Altona diagnostics GmbH, Hamburg, Germany) according to the manufacturers’ instructions on the LightCycler 96 (Roche Diagnostics, Basel, Switzerland). We tested serum from participants who presented within 7 days after symptom onset or urine within 14 days after symptom onset.^21^ We extracted RNA from 140 µL serum using QIAamp® RNA viral kit (Qiagen, Hilden, Germany). For urine, we mixed 500 µL with 500 µL cobas® PCR media kit (Roche Molecular Systems, Inc., Pleasanton, United States of America) before extracting RNA with the automated MagNa Pure purification system (Roche Molecular Systems, Inc.). For both serum and urine, we used 10 µL eluates for the RT–PCR assay. The PCR program consists of a RT reaction of 20 minute at 55 °C and a denaturation step of 2 minute at 95 °C, followed by 45 cycles of 15 second at 95 °C, 55 second at 58 °C and 15 second at 72 °C. We expressed RNA levels as threshold cycle values (C_t_-values), because a reference method for RNA quantification is not available. Any C_t_-value below 45 is defined as positive. We defined confirmed infection as a positive virus neutralization test result^22^ or a positive RT–PCR in any clinical sample.

Men who were 18 years or older, with a laboratory-confirmed symptomatic Zika virus infection and no history of immunosuppression were eligible to participate in the study. Staff members of the Institute of Tropical Medicine recruited men and recorded clinical and epidemiological data in a standardized case record form (Table 1). Two authors subsequently entered the data in a Microsoft Excel 2010 database (Microsoft Corp., Redmond, USA). For periodic monitoring of Zika virus RNA loads in semen, the participants were instructed to produce semen samples into a sterile cup by masturbation every week. Upon recruitment we provided the participants with materials needed for semen collection. The participants produced the sample at the institute or at home and sent the samples by regular mail at room temperature to the institute. We asked participants to produce semen samples until we obtained two negative results.

**Table 1:** Characteristics of men with confirmed Zika virus infection included in the prospective study on Zika virus kinetics in semen, Belgium, 2016–2017

Patient no.	Age, years	Travel destination	Date of symptom onset, 2016	Reported symptoms	Duration of illness (days)	Method of initial Zika virus diagnosis (type of sample)	Semen sample						
							First sample collected, days after symptom onset	No. of samples collected	Zika virus RNA detected	No. of days between symptom onset and first negative RT–PCR	Leukocyte count, cells/μL	Erythrocyte count, cells/μL	Sperm count, million/mL
1	45	Venezuela	6 February	Rash, fever, myalgia and headache	6	RT–PCR (urine)	20	6	Yes	58	960	922	8.8^d^
2	33	Haiti	6 February	Rash, fever, myalgia and haematospermia	7	RT–PCR (urine)	16	6	Yes	56	ND	ND	ND
3	38	Guadeloupe	15 July	Rash, myalgia and headache	3	RT–PCR (urine)	18	4	Yes	32	92	4	3.4^d^
4	54	Belize and Guatemala	1 August	Rash, fever, arthralgia, myalgia, fatigue and diarrhoea	7	RT–PCR (urine)	11^a^	13	Yes	103	180	280	36.8
5	28	Nicaragua	7 August	Rash, arthralgia, myalgia headache and diarrhoea	5	RT–PCR (urine)	14	18	Yes	144	102	0	25.2
6	55	Dominican Republic	15 July	Rash and fever	1	VNT (serum)	42	6	Yes	92	1850	72	N/A^b^
7	62	Cuba	30 November	Rash, fever and diarrhoea	4	RT–PCR (urine)	18	6	Yes	47^c^	40	20	0.4^d^
8	28	Guatemala and Nicaragua	12 December	Rash, fever, headache and conjunctivitis	6	RT–PCR (urine)	49	10	Yes	126	ND	ND	ND
9	44	Aruba and Curaçao	13 December	Rash, fever, arthralgia, myalgia, conjunctivitis, and fatigue	6	VNT (serum)	23	2	Yes	100	ND	ND	ND
10	46	Martinique	8 April	Rash and fever	4	VNT (serum)	26	2	No	N/A	252	4	24.1
11	30	Guadeloupe	2 June	Rash, fever, headache and diarrhoea	5	RT–PCR (serum and urine)	10	2	No	N/A	112	12	0.9^d^
12	46	Dominican Republic	4 June	Rash and fever	1	VNT (serum)	30	1	No	N/A	200	44	2.9^d^
13	48	Jamaica	17 July	Rash, fever, arthralgia, myalgia, conjunctivitis, and diarrhoea	5	RT–PCR (urine)	19	3	No	N/A	20	0	22.6
14	19	Guadeloupe	22 July	Rash, fever, arthralgia, myalgia and headache	10	RT–PCR (urine)	15	3	No	N/A	182	14	10.2^d^
15	65	Mexico	25 October	Rash and diarrhoea	3	VNT (serum)	42	2	No	N/A	ND	ND	ND

N/A: not applicable; ND: not determined; RNA: ribonucleic acid; RT–PCR: reverse-transcriptase polymerase chain reaction; VNT: virus neutralization test.

^a^ We managed to isolate the Zika virus from this sample by inoculation of C6/36 cells and Vero cells.

^b^ Patient was vasectomized.

^c^ Recurrence of Zika virus-RNA detection in semen after one negative RT–PCR result at day 32.

^d^ Oligospermia defined as a sperm count < 15 million sperms per mL.^23^

Notes: We used RealStar® Zika Virus RT–PCR kit (Altona diagnostics GmbH, Hamburg, Germany) for RNA detection on patient with active infection. VNT, a non-commercial test, was used for patients who presented at the clinic after the active viraemic phase.

If the laboratory staff received the first sample within an hour after production, they analysed the sperm, leukocyte and erythrocyte counts. Oligospermia was defined as a count of less than 15 million spermatozoa/mL.^23^ For the Zika virus RT–PCR analysis, laboratory staff transferred 140 µL semen into sterile Eppendorf tubes after liquefaction (30 to 60 minutes after ejaculation) and used QIAamp® RNA viral kit for RNA extraction. The RT–PCR was done as described above. Laboratory staff attempted to isolate the Zika virus from all initial semen samples by inoculating the supernatant of liquefied and centrifuged semen with confluent C6/36 cells growing in Eagle's minimal essential medium with 2% fetal bovine serum. When considerable virus-induced cytopathic effects were visible or after one week, the supernatant was passaged on to Vero cells and grown for one week. Laboratory staff monitored virus-induced cytopathic effect and confirmed such effects by RT–PCR.

### Statistical analysis

Because of the exploratory character of the study, we arbitrarily set the sample size at 20 participants. For each participant, we calculated the time from symptom onset until Zika virus RNA could no longer be detected in semen. We assessed the duration of Zika virus persistence in semen using the Kaplan–Meier estimator and the parametric Weibull regression models. All analyses were done in R, version 3.3.2 (R Foundation for Statistical Computing, Vienna, Austria).

### Ethical approval

We obtained ethical approval from the institutional review board at Institute of Tropical Medicine and the ethics committee of the Antwerp University Hospital, Belgium. We registered the protocol at ClinicalTrials.gov (NCT 02733796).

## Results

From February 2016 to May 2017, the institute recruited and followed-up 15 Caucasian men, who had travelled to countries with active vector-borne transmission of Zika virus (Table 1). We did not reach the projected sample size, because of the decline in number of patient with Zika virus infection. The participants’ median age was 45 years (range: 19–65). All men presented with rash; 12 (80%) had fever; eight (53%) had myalgia; six (40%) had headache or retro-orbital pain; five (33%) had arthralgia; and three (20%) had conjunctivitis. Six (40%) men reported diarrhoea and two (13%) described fatigue. The median duration of illness was 5 days (range: 1–10).

RT–PCR analyses confirmed Zika virus infection in 10 men, while virus neutralization tests confirmed infection in five men. All men consented to participate and 13 men completed the study; two patients did not provide enough numbers of semen samples for obtaining two consecutive negative results. Patient 9 provided only two semen samples (at 23 and 100 days after symptom onset) and patient 12 provided only one sample at 30 days after symptom onset, after which he withdrew (no reason given).

**Fig. 1:**
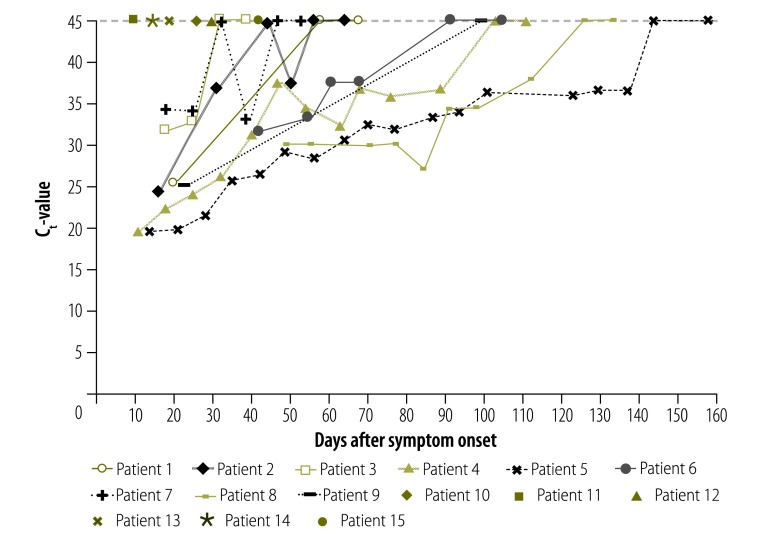
Detection of Zika virus RNA in semen from Belgian travellers, by days after symptom onset, 2016–2017

We detected Zika virus-RNA in the semen from nine men (60%), of which one man has had a successful vasectomy. For patients with RNA in semen, the median number of samples analysed was 6 (range: 2–18). For men with positive RT–PCR result, the median time to collection of the first semen sample was shorter than for men with a negative result, though not significantly (18 days after symptom onset; range: 10–49 versus 23 days after symptom onset; range: 10–42). The longest duration before obtaining the first negative semen RT–PCR result was 144 days after symptom onset, with the last sample testing positive at 137 days after symptom onset (Fig. 1). For patient 7 we observed viral RNA recurrence in semen after a single negative RT–PCR result (Fig. 1).

RNA: ribonucleic acid; C_t_: threshold cycle.

Notes: We used RealStar® Zika Virus reverse-transcriptase polymerase chain reaction kit (Altona diagnostics GmbH, Hamburg, Germany) for RNA detection. The dashed line represents the cut-off and a C_t_-value below 45 is considered positive (detection of RNA) and a C_t_-value of 45 is defined as negative. For patients 10, 12, and 15, we did not detect RNA in any sample. Two patients did not complete the study, i.e. obtaining two negative results: patient 9 provided only two semen samples and patient 12 provided only one sample.

**Fig. 2:**
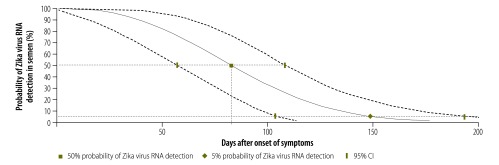
Estimated time to loss of Zika virus RNA detection in semen, Belgium, 2016–2017

CI: confidence interval; RNA: ribonucleic acid.

Notes: The Weibull distribution of probability of Zika virus RNA detection in semen assumes 100% probability at the start. We only included participants with detectable Zika virus RNA in semen (*n* = 9). The solid line represents the median probability of survival; the dashed lines represent the upper and lower margins of the 95% confidence interval.

The Weibull distribution curve showed that the median loss of RNA detection in semen occurred at 83 days after symptom onset (95% CI: 57−108). At 149 days after symptom onset (95% CI: 104−194) RNA could no longer be detected in the semen of 95% of the men (Fig. 2).

We managed to isolate the Zika virus from one semen sample, collected on 11 days after symptom onset and with a RT–PCR C_t_-value of 19.6. Cytopathic effect appeared only in Vero cells after passage on the C6/36 culture.

For 11 patients, the first collected semen sample was available for microscopic analysis. We detected leukocytes in all samples and erythrocytes in nine samples; these included five Zika virus positive and four negative samples (Table 1). For one participant, we found macroscopic haematospermia and detected viral RNA in his semen. After excluding the vasectomized man (patient 6), six out of 10 participants for whom microscopic analysis of semen was available, had oligospermia. Three of these men had Zika virus positive samples and three had negative samples. Out of the six men with oligospermia, five had reported fever. Only one individual completed the follow-up to monitor normalization of sperm counts.

## Discussion

Here we report on the frequency and persistence of Zika virus in semen after acute symptomatic infection in Belgian men who had travelled to the Americas during the 2016 Zika virus outbreak. In 60% of the men with confirmed Zika virus infection, we also detected Zika virus RNA in the semen. Several studies have reported a similar proportion of detection, both in endemic settings^13^^,^^17^^,^^18^ and in travellers.^19^ For the six participants with no detectable RNA in semen, we cannot exclude early infection of the male reproductive organs, since others have reported short-lasting presence of Zika virus RNA in semen, as short as 1 day after symptom onset.^17^ The median time to the first sampling of semen from these six men was 23 days after symptom onset, which suggest that the Zika virus may have already been eliminated from the semen. Therefore, we as well as other researchers might have underestimated the proportion of men with virus in their semen.

Persistence of Zika virus RNA in semen after acute infection appears common. In our study, sequential semen samples showed decreasing level of RNA until the virus became undetectable, confirming previous findings.^13^^,^^17^^,^^19^ However, our data showed almost two months longer clearance time than the cohort studies from endemic settings.^13^^,^^17^^,^^18^ We cannot ensure that a difference existed in seminal viral persistence between travellers and endemic populations. Both our and other studies may have underestimated the duration of viral shedding in patients with longer semen sampling intervals or in patients having only one negative RT–PCR result.^13^^,^^17^^,^^19^ We could exclude differences in diagnostic sensitivity since we used the same RT–PCR assays as two of the other studies.^17^^,^^18^ It is also unlikely that the differences can be attributed to ethnic background of the host, as the proportion of infected semen samples did not differ across our cohort and the endemic studies. One possible explanation could be the difference in natural acquired immunity or vaccination status between men residing in endemic areas and travellers. People living in endemic areas are exposed to other circulating flaviviruses, such as dengue and chikungunya viruses, which may affect their ability to clear the virus. In our cohort, we could not assess previous exposure to dengue virus, since the antibody detection assays cross-react with the Zika virus antibodies.

The decline of Zika virus-RNA levels in semen observed in our patients suggests elimination of the virus. In only one patient, we could detect viral RNA after one negative RT–PCR result, but who subsequently had two consecutive negative samples that concluded follow-up. This finding demonstrates the need for two consecutive negative results, since the possibility of viral recurrence cannot be excluded.

Almost all men who had their semen analysed showed macroscopic or microscopic haematospermia. Other studies have also investigated haematospermia following Zika virus infection, but the symptom has not been consistently found.^11^^,^^14^^,^^16^^,^^24^ The observation of inflammatory cells in semen, irrespective of Zika virus RNA detection, may indicate some degree of tissue damage to the male reproductive tract in the majority of infected men. The presence of erythrocytes, leucocytes and oligospermia in semen could be a sign of inflammation and disruption of the tight-junctions between Sertoli cells that form the blood-testis barrier.^25^ In a mouse model, Zika virus infection resulted in histological injury to Sertoli and Leydig cells and to the lumen of the epididymis, and the infection was associated with reduced levels of inhibin β and testosterone.^25^ Recently, a human study demonstrated that men with active Zika virus infection had increased follicle-stimulating hormone concentrations, while they had decreased inhibin β and testosterone concentrations, and median sperm counts.^18^ The presumed target cells for Zika virus replication in the male reproductive tract are therefore likely to include the seminiferous epithelium. Interestingly, the duration of Zika virus shedding in semen in our cohort may coincide with the time required for spermatogonial renewal, differentiation and proliferation in humans (reported to be 74 days; 95% CI: 69–80).^26^

Some of the participants had oligospermia, which could result from inflammatory destruction of the seminiferous epithelium directly, from the fluctuation of reproductive hormone concentrations or from the febrile state.^18^

As reported previously, presence of viral RNA in the ejaculate of the vasectomized participant suggests that the Zika virus does not only infect spermatogonia, but also tissues distal of the vasectomy site.^27^^,^^28^

Isolation of the virus is regarded as a proxy for infectivity. However, we were only able to isolate the Zika virus from one of our patients’ semen. The failure to isolate the virus from the other patients could be an indication of viral degradation, although others have demonstrated Zika virus replication competence in semen up to 69 days after symptom onset.^28^ Lower viral loads or laboratory conditions may explain why virus isolation was not more successful.^29^^,^^30^ However, as long as the virus can be detected in semen by RT–PCR, we do not exclude that the virus can be sexually transmitted.

Zika virus in semen may become the major transmission route in areas where arthropod vectors do not thrive. However, mathematical modelling suggests that sexual transmission alone is not likely to drive or sustain a Zika virus outbreak in absence of a suitable vector population.^31^ Concerns remain that sexual transmission of Zika virus to pregnant women may increase the risk of poor neonatal outcomes in addition to vector-borne infection alone. In mouse models, vaginal infection during pregnancy led to restriction of fetal growth and to fetal brain infection.^32^^,^^33^

Our study has at least three limitations. First, we had to discontinue the recruitment of participants before reaching the projected sample size, because incidence of Zika virus infection had declined by May 2017.^34^ The relatively small sample size limits the generalizability of our results. Second, the follow-up period after obtaining two negative RT–PCR results in semen samples was relatively short. This time may have been too short to detect recurrence of RNA in semen. Third, we could not ascertain the recovery of sperm counts in men with oligospermia. Production of fresh samples suitable for microscopic semen analysis at the study site, proved too demanding for the participants.

The findings presented here emphasize that further studies are needed to increase our understanding of the host-pathogen relationship and implications of Zika virus infection for reproductive function. Pending new evidence, we recommend the use of RNA detection assays for semen of returning travellers with confirmed Zika virus infection, especially for couples planning a pregnancy. To reduce the risk of sexual transmission of the virus, our findings highlight that health professionals should advice patients, even vasectomized men, about the current recommendations from the World Health Organization and United States Centers for Disease Control and Prevention.^35^^,^^36^
